# Tolerance and autoimmunity in the liver

**DOI:** 10.1007/s00281-022-00952-6

**Published:** 2022-07-05

**Authors:** Christoph Schramm, Ye H. Oo, Ansgar W. Lohse

**Affiliations:** 1 Ist Dept of Medicine, University Medical Center Hamburg-Eppendorf, Martinistr. 52, 20246 Hamburg, Germany; 2 Martin Zeitz Center for Rare Diseases, University Medical Center Hamburg-Eppendorf, Hamburg, Germany; 3 Hamburg Center for Translational Immunology (HCTI), Hamburg, Germany; 4 European Reference Network On Hepatological Diseases (ERN RARE-LIVER), Hamburg, Germany; 5 Liver Transplant Unit, Centre for Rare Disease, Birmingham & Centre for Liver and Gastro Research, Institute of Immunology and Immunotherapy, Queen Elizabeth Hospital, University Hospital of Birmingham, University of Birmingham, Birmingham, UK

Autoimmune diseases arise from the failure of immune tolerance towards self-antigens. In the liver, maintenance of immune tolerance is particularly challenging. The liver filters all blood from the intestinal tract and is thus exposed to an infinite number of nutritional and microbial antigens. Additionally, the liver harbours the large mucosal surface of the biliary system which is colonized with microbiota and exposed to environmental factors. Together with neo-antigens arising from hepatic metabolism, the liver therefore is constantly challenged to maintain tolerance against harmless antigens and to rise immune responses against harmful environmental exposure. It is presumably for this reason that the liver microenvironment—under normal circumstances—is a particularly potent inducer of immune tolerance within the organ itself, but also systemically. For example, targeting liver sinusoidal endothelial cells using nanoparticles revealed that these cells can be harnessed to induce tolerance to the central nervous system autoantigen myelin basic protein, which can prevent the development of experimental autoimmune encephalomyelitis [1]. While the tolerogenic liver microenvironment prevents immune attack in the healthy liver, it presumably also provides a survival benefit for cancer cells and hepatotropic viruses by preventing their elimination by the immune system.

In spite of its tolerogenic potential, the liver can fall victim to three major autoimmune diseases for which curative therapies are lacking: autoimmune hepatitis (AIH), primary biliary cholangitis (PBC) and primary sclerosing cholangitis (PSC). Therefore, these diseases are leading indications for liver transplantation. Understanding the making and breaking of liver tolerance will enable the development of novel and effective immunotherapies. These insights are not only relevant for the treatment of the autoimmune liver diseases, as understanding these mechanisms can be key to develop tools to manipulate systemic immunity and to treat autoimmune diseases outside of the liver and bile ducts.

Within this special issue of Seminars in Immunopathology, we highlight recent advances in the understanding of mechanisms governing tolerance and autoimmunity in the liver. We focus on research areas with a clear potential for novel therapeutic strategies. Genetic risk and environmental exposures both contribute to the development of autoimmunity in the liver. Among the autoimmune liver diseases, genetic risk is highest for PBC. Genome-wide association studies have highlighted the potential contribution of the immune system, and specifically the adaptive immune system for disease pathogenesis. However, functional studies on polymorphisms associated with disease risk are largely lacking. Summarizing how genetic risk contributes to autoimmune liver disease, Ellinghaus gives an outlook on how genetic analyses can improve our understanding of disease mechanisms in the future. This also includes the identification of autoantigens, information on which is largely lacking [2]. Autoantibodies are characteristic of AIH and PBC and have recently been associated with PSC prognosis as well. Additionally, AIH is characterized by selective elevation of serum Immunoglobulin G levels and by infiltration of the liver by B cells and plasma cells. Few case series suggested that B cell depletion therapy may improve disease activity in difficult to treat AIH patients. Schultheiß and colleagues discuss whether B cells in AIH are only bystanders or central players of disease pathogenesis and thus therapeutic targets in AIH [3]. Mucosa-associated invariant T (MAIT) cells are highly abundant in human liver. Their invariant T cell receptor recognizes bacterial metabolites of B vitamins. MAIT cells also respond to a number of cytokines expressed in liver inflammation. It can be suggested that MAIT cells play an important role at the mucosal barrier of bile ducts, acquiring either regenerative or pro-inflammatory and pro-fibrogenic potential according to the environmental exposure. Mehta et al. summarize the current knowledge on MAIT cells in the liver and how different ways of stimulation may shape their function within human liver [4]. Targeting cytokines may be an attractive therapeutic option independent from specific cell types. TNF is a central pro-inflammatory cytokine in autoimmune diseases including AIH. It has recently been shown that neutralizing TNF can be used to effectively control difficult to treat AIH patients. On the other hand, anti-TNF treatment can induce liver inflammation in patients with extrahepatic immune-mediated diseases such as inflammatory bowel disease. It is therefore essential to dissect the role of TNF in immune homeostasis and autoimmunity in the liver in order to develop precise and well-tolerated therapies. Tiegs et al. summarize current knowledge on TNF, signaling through TNF receptors I and II and how this could lead to novel therapies of autoimmune liver diseases [5]. Regulatory T cells (Treg) belong to the body’s armamentarium to maintain and restore peripheral immune tolerance, both in an antigen-specific and -unspecific way. Targeting Treg numbers and function currently is intensely investigated as therapy for many autoimmune diseases. Deficiencies of Treg have been reported in autoimmune liver diseases, but remain a matter of debate depending on disease and tissue analyzed. Oo and colleagues have recently reported the migration of ex vivo expanded Treg into the liver of people with AIH [6] and here review the challenges and opportunities in achieving effective Treg therapy in autoimmune liver diseases as a promising future tool to improve treatment without harmful side effects [7].

As mentioned above, the liver’s tolerogenic potential can be strengthened to treat hepatic autoimmunity, but it may also be exploited to induce systemic immune tolerance. One of the hepatic cell types with a high tolerogenic potential are the hepatic sinusoidal endothelial cells, that form the lining of liver sinusoids and are thus exposed first line to environmental factors entering the liver via the portal vein and thus the intestinal tract. Gottwick and colleagues therefore focus in their review on how the liver can be harnessed to induce antigen-specific immune tolerance systemically [8]. Microbial products and microbiota themselves enter the liver via the portal vein. In addition, the portal tracts of the liver are constantly exposed to the luminal and mucosal microbiota present in the biliary system. It is now clear that autoimmune liver disease patients harbour a microbiota composition that is different from healthy people and different between the diseases. Additionally, it has recently been shown that bile fluid harbours a rich microbiota composition that is different in people with PSC [9]. In this special issue, Liwinski et al. amend this concept and highlight current evidence and concepts on how the intestinal and biliary microbiome affect autoimmune liver disease [10]. Along this line, Nakamoto et al. comprehensively review the current understanding of IL-17 producing CD4 + T cells (Th17) in autoimmune liver diseases and specifically in PSC. IL-17 plays an important role in pathogen defence, e.g. by recruiting neutrophil granulocytes. On the other hand, IL-17 has emerged as a promising treatment target in several autoimmune diseases outside of the liver. Thus, Nakamoto and colleagues have recently described a pathogenetic role of microbiota-induced Th17 cells in PSC pathogenesis [11]. Here, they now review the current understanding of the dual role of IL-17 and Th17 cells in balancing autoimmunity and pathogen defence [12]. Biliary epithelial cells form the mucosal barrier of bile ducts and are targets of autoimmune inflammation in PBC and PSC. They may not only be targets, but also active contributors to liver inflammation. Their senescence-associated secretory phenotype has been shown to promote periductal inflammation. Trussoni et al. summarize current understanding of cellular senescence and its role as a driver of immunopathology and therapeutic target in the cholangiopathies [13]. To conclude this special issue, Bertolini and colleagues review the central role of bile acids and their receptors as modulators and therapeutic targets in liver inflammation [14]. Indeed, bile acid derivatives are the only licenced treatment options for PBC and drugs targeting the Farneoid X Receptor (FXR), the transcription factor central to the regulation of bile acid homeostasis, are being studied for the treatment not only of PBC and PSC, but also for other liver diseases such as non-alcoholic (or metabolic associated) steatohepatitis. Additionally, the uptake of bile acids is targeted for treatment of hereditary cholestatic liver diseases, underlining the important role bile acids play in immune mediated and autoimmune liver diseases.

Taken together, in this special issue, we highlight some of the topics important for the development of future therapies for autoimmune liver diseases. Some of these concepts have started to enter the clinic and care of patients, others are being developed to offer more targeted and better tolerated therapies for patients with autoimmune liver diseases. Current treatment, if available, often is associated with severe side effects, ineffective in a considerable proportion of patients and unable to induce cure. With increasing knowledge of the mechanisms underlying tolerance and autoimmunity in the liver, there is realistic hope to improve the care of these difficult to treat diseases in the future.
